# *Trypanosoma cruzi* Infection and Endothelin-1 Cooperatively Activate Pathogenic Inflammatory Pathways in Cardiomyocytes

**DOI:** 10.1371/journal.pntd.0002034

**Published:** 2013-02-07

**Authors:** Ricardo S. Corral, Néstor A. Guerrero, Henar Cuervo, Núria Gironès, Manuel Fresno

**Affiliations:** 1 Servicio de Parasitología-Chagas, Hospital de Niños Ricardo Gutiérrez, Buenos Aires, Argentina; 2 Centro de Biología Molecular Severo Ochoa, Consejo Superior de Investigaciones Científicas, Universidad Autónoma de Madrid, Cantoblanco, Madrid, Spain; 3 Instituto de Investigación Sanitaria Princesa, Hospital Universitario de La Princesa, Madrid, Spain; National Institutes of Health, United States of America

## Abstract

*Trypanosoma cruzi*, the causative agent of Chagas' disease, induces multiple responses in the heart, a critical organ of infection and pathology in the host. Among diverse factors, eicosanoids and the vasoactive peptide endothelin-1 (ET-1) have been implicated in the pathogenesis of chronic chagasic cardiomyopathy. In the present study, we found that *T. cruzi* infection in mice induces myocardial gene expression of cyclooxygenase-2 (*Cox2*) and thromboxane synthase (*Tbxas1*) as well as endothelin-1 (*Edn1*) and atrial natriuretic peptide (*Nppa*). *T. cruzi* infection and ET-1 cooperatively activated the Ca^2+^/calcineurin (Cn)/nuclear factor of activated T cells (NFAT) signaling pathway in atrial myocytes, leading to COX-2 protein expression and increased eicosanoid (prostaglandins E_2_ and F_2α_, thromboxane A_2_) release. Moreover, *T. cruzi* infection of ET-1-stimulated cardiomyocytes resulted in significantly enhanced production of atrial natriuretic peptide (ANP), a prognostic marker for impairment in cardiac function of chagasic patients. Our findings support an important role for the Ca^2+^/Cn/NFAT cascade in *T. cruzi*-mediated myocardial production of inflammatory mediators and may help define novel therapeutic targets.

## Author Summary

Chronic cardiomyopathy is the most common and severe manifestation of human Chagas' disease, caused by the protozoan parasite *Trypanosoma cruzi*. Among diverse inflammation-promoting moieties, eicosanoids and the vasoactive peptide endothelin-1 (ET-1) have been implicated in its pathogenesis. Nevertheless, the link between these two factors has not yet been identified. In the present study, we found that *T. cruzi* infection induces gene expression of ET-1 and eicosanoid-forming enzymes in the heart of infected mice. We also demonstrated that HL-1 atrial myocytes respond to ET-1 stimulus and *T. cruzi* infection by induction of cyclooxygenase-2 through activation of the Ca^2+^/calcineurin/NFAT intracellular signaling pathway. Moreover, the cooperation between *T. cruzi* and ET-1 leads to overproduction of eicosanoids (prostaglandins E_2_ and F_2α_, thromboxane A_2_) and the pro-hypertrophic atrial natriuretic peptide. Our results support an important role for NFAT in *T. cruzi* plus ET-1-dependent induction of key agents of pathogenesis in chronic chagasic cardiomyopathy. Identification of the Ca^2+^/calcineurin/NFAT cascade as mediator of cardiovascular pathology in Chagas' disease advances our understanding of host-parasite interrelationship and may help define novel potential targets for therapeutic interventions to ameliorate or prevent cardiomyopathy during chronic *T. cruzi* infection.

## Introduction

Chagas' disease, caused by the infection with the protozoan parasite *Trypanosoma cruzi*, constitutes the major cause of infectious heart disease in Latin America. It is estimated that 10 million people are infected with *T. cruzi* in the Central and South America, 100–120 million are at potential risk of infection and around 50,000 new cases occur each year [1]. In humans, an acute phase displays frequently as a non-apparent form with a few or no symptoms. Thereafter, the patients enter into an asymptomatic, indeterminate stage, which lasts throughout life in the majority of infected subjects. The remaining 20–30% of chronically infected individuals develop cardiac or digestive complications, typically years or decades after infection. Chronic cardiomyopathy is the most common and severe manifestation of human Chagas' disease, causing congestive heart failure, arrhythmias and conduction abnormalities, which often lead to stroke and sudden death. This type of dilated cardiomyopathy is associated with chronic inflammation and fibrosis, cardiac hypertrophy and thrombo-embolic events [2].

Compromised microcirculation, caused by *T. cruzi* infection, involves endothelial alterations, vasospasm, reduced blood flow and focal ischemia [3]. Cardiovascular production of vasoactive mediators has been implicated in the pathogenesis of the vasculopathy seen in chagasic heart disease [4]. Among other vasculitis-promoting factors, *T. cruzi* infection triggers myocardial overexpression and increased plasma levels of endothelin-1 (ET-1) in mice and chronic chagasic patients, which correlate with heart dysfunction [5], [6]. A bulk of evidence supports the participation of this vasoactive peptide, produced by myocardial and endothelial cells among others, in Chagas' disease pathogenesis [4], [5], [7], [8]–[10]. ET-1 activity may result in vascular injury, cardiac remodeling and enhanced liberation of inflammatory agents [11].

Endothelin-1 is involved in different signaling pathways that include increase in intracellular calcium levels ([Ca^2+^]_i_) and ERK1/2 activation leading to expression of cyclin D1 and inflammation-linked genes, all of them contributing to *T. cruzi*-mediated cardiac pathology [12], [13]. Moreover, ET-1 has been shown to induce cell hypertrophy in primary cultures of rat cardiomyocytes through a calcineurin (Cn)/nuclear factor of activated T cells (NFAT)-dependent mechanism [14], [15]. The NFAT family includes four ‘classical’ members displaying a high degree of homology: NFATc1-4, each of which is expressed in heart tissue [16]. NFAT exists in a highly phosphorylated form in the cytoplasm, which translocates into the nucleus upon dephosphorylation by the phosphatase Cn in response to increases in [Ca^2+^]_i_, where it binds to enhancer elements of downstream genes leading to transcriptional activation [17].

One of the NFAT target genes associated with inflammation is cyclooxygenase-2 (COX-2), the inducible enzyme that catalyzes the rate-limiting step in prostanoid biosynthesis [18]–[20]. ET-1 is able to stimulate protein expression of COX-2 and prostacyclin release in cardiomyocytes [21]. In addition, experimental murine infection with *T. cruzi* has been shown to raise the number of cardiac cells positive for COX-1 and COX-2, as well as the circulating levels of cyclooxygenase metabolites [22], [23]. Both host- and parasite-derived prostaglandins (PG) and thromboxane A_2_ (TXA_2_) are key regulators of pathogenesis during *T. cruzi* infection [24]. Remarkably, ET-1 stimulation of cardiac myocytes also results in NFATc4-dependent up-regulation of hypertrophy response genes such as atrial natriuretic peptide (ANP) and B-type natriuretic peptide (BNP) [25], [26], potential markers of myocardial compromise in Chagas' disease [27], [28].

Although ET-1 and eicosanoids have been proposed to play a role in Chagas' disease pathogenesis, the link between them has not yet been addressed. Thus, we have examined the regulation of *Cox2* expression and activity by the combined effect of ET-1 and *T. cruzi* infection of cardiomyocytes. Our results show that induction of *Cox2* expression by ET-1 plus *T. cruzi* in HL-1 atrial myocytes requires activation of the Ca^2+^/Cn/NFAT pathway. NFAT is translocated to the nucleus upon stimulation with the peptide and subsequent infection where it binds to NFAT response elements in the promoter region of *Cox2* that are essential for transcriptional induction of the gene. Moreover, trypomastigote infection of ET-1-pre-treated HL-1 cardiomyocytes significantly enhanced production of eicosanoids and ANP by these cells. These findings demonstrate the participation of NFAT in [*T. cruzi*+ET-1]-mediated induction of genes involved in the pathogenesis of chronic Chagas' heart disease.

## Materials and Methods

### Ethics statement

This study was carried out in strict accordance with the recommendations of Spanish Legislation and the European Council Directive from the Convention for the Protection of Vertebrate Animals Used for Experimental and Other Scientific Purposes. All mice were maintained under pathogen-free conditions in the animal facility at the Centro de Biología Molecular, Universidad Autónoma de Madrid (Madrid, Spain). The animal protocol was approved by the Comité de Ética de la Investigación de la Universidad Autónoma de Madrid. Animals had free access to food and water and were handled in compliance with European codes of practice. Mice were euthanized in a CO_2_ chamber, and all efforts were made to minimize suffering.

### Cell culture, primary cardiomyocytes and infection

Mouse HL-1 cardiomyocytes were plated onto gelatin/fibronectin pre-coated flasks and cultured in Claycomb medium (Sigma-Aldrich) supplemented with 10% fetal calf serum, 100 U/ml penicillin, 100 µg/ml streptomycin and 2 mM L-glutamine as previously described [29]. Primary cardiomyocytes were isolated from BALB/c mice and cultured according to standard protocols [30]. HL-1 and primary cardiomyocytes were seeded in 6- (5×10^5^/well) or 24- (1×10^5^/well) well plates and infected with *T. cruzi* trypomastigotes (cell∶parasite ratio 1∶5), Y strain, routinely propagated in Vero cells. In some experiments, cell cultures were starved for 18 h and then treated with recombinant murine interferon-γ (25 U/ml IFN-γ, R&D Systems), 1 µg/ml lipopolysaccharide (LPS, Sigma-Aldrich) or 0.3 nM ET-1 (Sigma-Aldrich) for 2 h before infection. Endotoxin level in the ET-1 batch was <1 EU/mg, as determined using a *Limulus* amoebocyte lysate analysis kit (Whittaker Bioproducts). Plates were rinsed to remove free parasites and further incubated in complete medium at 37°C, 5% CO_2_ for the indicated times.

### *In vivo* infection

Young adult (6- to 8-wk-old) C57BL/6 mice were purchased from Charles River Laboratories. For infection experiments, 2×10^3^ blood trypomastigotes (Y strain) per mouse were inoculated by intraperitoneal injection as described [31], keeping a group of non-infected mice. Age-matched BALB/c mice were infected in parallel. Parasitemia levels were checked every 2 days by direct inspection and counting parasites in a 5 µl drop of tail vein blood. Weekly during one month post-infection, groups of 3 mice were euthanized in a CO_2_ chamber, and blood and various tissues were collected. Samples were processed for RNA or histological analysis.

### RNA isolation, reverse transcription and polymerase chain reaction (PCR)

Total RNA was extracted from HL-1 cells and mouse heart tissue by using Trizol reagent (Invitrogen) according to the manufacturer's instructions. First-strand cDNA was prepared by incubation of 1 µg of total RNA with murine leukemia virus reverse transcriptase and random hexamer oligonucleotides (Bio-Rad Laboratories) at 40°C for 45 min. Then, 5 µl of the reaction products was amplified by PCR with 1.25 U of *Taq* DNA polymerase (Invitrogen). PCR amplification consisted of 94°C for 45 s for denaturation, 60°C for 45 s for annealing, and 72°C for 45 s for extension, performed for 30 cycles. The sense and antisense primers used for murine *Cox2* were: 5′-tcctcctggaacatggactc-3′ and 5′-gctcggcttccagtattgag-3′, respectively [32]. Aliquots of 10 µl of the PCR products were electrophoresed in a 1.6% agarose gel containing ethidium bromide.

### Real-time PCR of infected heart tissue

Quantitative real-time RT-PCR analysis was performed using the High Capacity cDNA Archive Kit (Applied Biosystems), and amplification of different murine genes (*Cox2*, *Cox1*, *Tbxas1*, *Nppa*, *Edn1* and ribosomal 18S) was performed in triplicate with the use of TaqMan MGB probes and the TaqMan Universal PCR Master Mix (Life Technologies) on an ABI Prism 7900 HT instrument (Applied Biosystems), as reported previously [31]. Quantification of gene expression was calculated using the comparative threshold cycle (C*t*) method, normalized to the ribosomal 18S control and efficiency of the RT reaction (relative quantity, 2^−ΔΔC^ *T*).

### Histological and immunohistochemical analysis of heart

Cardiac tissues from mice were placed after been cut in two pieces in 10% neutral buffered formalin for at least 4 h at room temperature followed by overnight incubation in 70% ethanol. Samples were them embedded in paraffin (Tissue Embedding Station Leica EG1160), and 5-µm tissue sections were prepared using a motorized Microtome Leica RM2155. Samples were deparaffinized and rehydrated using a Tissue Processing Station Leica TP1020. Slides were stained using the Masson's trichrome staining and mounted permanently in Eukkitt's quick hardening mounting system medium (Biochemika, Fluka Analytical). The sections were analyzed in a Leica DMD 108 microscope (Leica Microsystems, Germany). For immunohistochemical studies, myocardial sections were deparaffinized by routine procedures and analyzed using anti-murine COX-2 rabbit polyclonal antibody (Abcam) and biotinylated swine antiserum to rabbit immunoglobulin (Dako), following a procedure previously described [33].

### Immunoblot analysis

Immunoblotting was carried out as described elsewhere [19]. Cardiac cells were disrupted and solubilized extracts (20 µg) were separated in 6% (only for analysis of NFAT translocation to the nucleus) or 10% sodium dodecyl sulfate-polyacrylamide gels, and transferred to nitrocellulose filters. After blocking for 2 h with 5% non-fat dried milk in Tris-buffered saline containing 0.1% Tween-20, the membranes were probed 2 h at 37°C with murine monoclonal antibodies against COX-2 (diluted 1∶250 in blocking buffer, BD Biosciences), α-tubulin (1∶1000, Sigma-Aldrich), and with rabbit polyclonal antibodies against NFAT (c1 to c4 isoforms, 1∶200, Santa Cruz Biotechnology), prostaglandin E synthase-2 (microsomal, 1∶500), thromboxane synthase (1∶500, Cayman) and prostaglandin F synthase/AK31C3 (1∶2,000, ProSci). The filters were washed and incubated with the corresponding secondary antibody linked to horseradish peroxidase at 1∶10,000 dilution, and the stained bands were visualized by a chemiluminescent peroxide substrate (Amersham Pharmacia).

### Plasmid constructs

*Cox2* promoter constructs spanning from −1796 (P2-1900-LUC) and −170 (P2-274-LUC) to +104 bp relative to the transcription start site of the human *Cox2* gene and the P2-274-LUC plasmid with binding sites for NFAT, or AP-1, or both mutated were described [19]. The pSH102CD418 expression vector derives from pBJ5 and encodes an NFATc1 deletion mutant (1–418) that functions as a dominant negative for all NFAT isoforms [34].

### Transfection and luciferase assays

HL-1 cells were transfected by Lipofectamine (Invitrogen) as described [19]. Briefly, exponential growing cells (2×10^5^/well) cultured in 24-well plates were incubated for 3 h at 37°C with a mixture of 0.5–1 µg of the corresponding reporter plasmid and Lipofectamine-containing Opti-MEM (Invitrogen). The total amount of DNA in each transfection was kept constant by using the empty expression vectors. Complete medium was then added to cells and incubated at 37°C for additional 16 h. Transfected cells were exposed to different stimuli (0.3 nM ET-1, or phorbol 12-myristate 13-acetate -PMA- plus A23187 calcium ionophore -Ion-, Sigma-Aldrich) and/or *T. cruzi*-infected as indicated. In some experiments, FK506 (100 ng/ml, Sandoz Ltd., Tokyo, Japan) was added for 1 h. Then, cells were harvested and lysed. Luciferase activity was determined by using a luciferase assay system (Promega) with a luminometer Monolight 2010 (Analytical Luminescence). Transfection experiments were performed in triplicate. Data of luciferase activity are presented as fold induction (observed experimental relative luciferase units (RLU)/basal RLU in absence of any stimulus). Results were normalized for extract protein concentrations measured with a Bradford assay kit (Pierce, Thermo Fisher Scientific).

### Intracellular calcium measurements

Agonist-induced changes in [Ca^2+^]_i_ were detected using the Ca^2+^-sensitive dye Fura-2/AM as described [35]. Briefly, cell monolayers at 80% confluence were trypsinized, washed and then loaded with 1 µM Fura-2/AM under continuous stirring for 30 min at 37°C. The cells (2×10^6^/ml) were exposed to 0.3 nM ET-1 and/or infected with *T. cruzi* trypomastigotes (cell∶parasite ratio 1∶5), and placed in an Aminco Bowman Series 2 spectrofluorometer (Thermo). Uninfected cultures were used as controls. At the indicated times, the fluorescence signal of Fura-2 was recorded, with excitation and emission at 340 and 510 nm, respectively.

### Electrophoretic mobility shift assay (EMSA)

Nuclear extracts were prepared from ET-1-treated and/or *T. cruzi*-infected HL-1 cells as described [36] with minor modifications. Purity of fractions was proven by analyzing cytoplasmic and nuclear marker proteins including α-tubulin (cytoplasmic), and topoisomerase IIβ and c-jun (nuclear). In brief, 5 µg of nuclear protein was incubated with 1 µg of poly(dI–dC) DNA carrier in DNA binding buffer (10% (wt/vol) polyvinylethanol, 12.5% (vol/vol) glycerol, 50 mM Tris, pH 8, 2.5 mM dithiothreitol, 2.5 mM ethylenediaminetetraacetic acid) for 30 min at 4°C. Then, 10^5^ counts per minute (c.p.m.) (10^8^ c.p.m./µg) of the ^32^P-labeled double-stranded oligonucleotide (2 µg) were added, and the reaction was incubated at room temperature for 30 min. A synthetic oligonucleotide containing the NFAT consensus sequence 5′-gggtggggtggggaaagccgaggcgga-3′ (nucleotides −98 to −73) in the rat Cox-2 promoter was used as probe/competitor in EMSAs. For competition experiments, a 50-fold molar excess of unlabeled oligonucleotide was added before the addition of the probe. Supershift assays were performed by incubating nuclear extracts with either normal rabbit IgG or anti-NFATc4 antibody for 15 min at 4°C before the addition of the probe. DNA-protein complexes were resolved by electrophoresis in 4% non-denaturing polyacrylamide gels and were subjected to autoradiography.

### Measurements of metabolites

For eicosanoid measurements, HL-1 cells were maintained for 12 h in culture medium supplemented with 0.5% fetal calf serum, then pre-treated or not with 10 µM indomethacin (Sigma-Aldrich) or 10 µM NS-398 (Alexis) for 1 h, and further stimulated with 0.3 nM ET-1 for 2 h. After treatment, cardiomyocytes were infected with *T. cruzi* trypomastigotes for 24 h. At that time, media supernatants were collected and analysed for PGE_2_, PGF_2α_ and TXB_2_ by ELISA (Cayman) according to manufacturer's specifications. In addition, eicosanoid levels were determined by ELISA in the sera from both uninfected and *T. cruzi*-infected C57BL/6 mice at 21 days of infection.

For ANP measurements, 24-h supernatants from ET-1-stimulated and/or *T. cruzi*-infected HL-1 cells, as well as serum specimens from both uninfected and *T. cruzi*-infected mice, were analyzed by ELISA (Kamiya Biomedical) following the instructions of the supplier.

For ET-1 measurements, the sera from uninfected and *T. cruzi*-infected mice were analyzed by ELISA (Phoenix Pharmaceuticals), according to the manufacturer's guidelines.

### Statistical analysis

Statistical analysis was performed by using GraphPad Prism 5.0 software. Arithmetics means and standard error of the means (s.e.m.) were calculated. Significant differences among groups were made by using the one-way analysis of variance test followed by Tukey's test. A difference between groups of *P*<0.05 was considered significant.

## Results

### *Trypanosoma cruzi* infection induces the expression of markers of cardiac damage and eicosanoid enzymes in the heart

**Figure 1:**
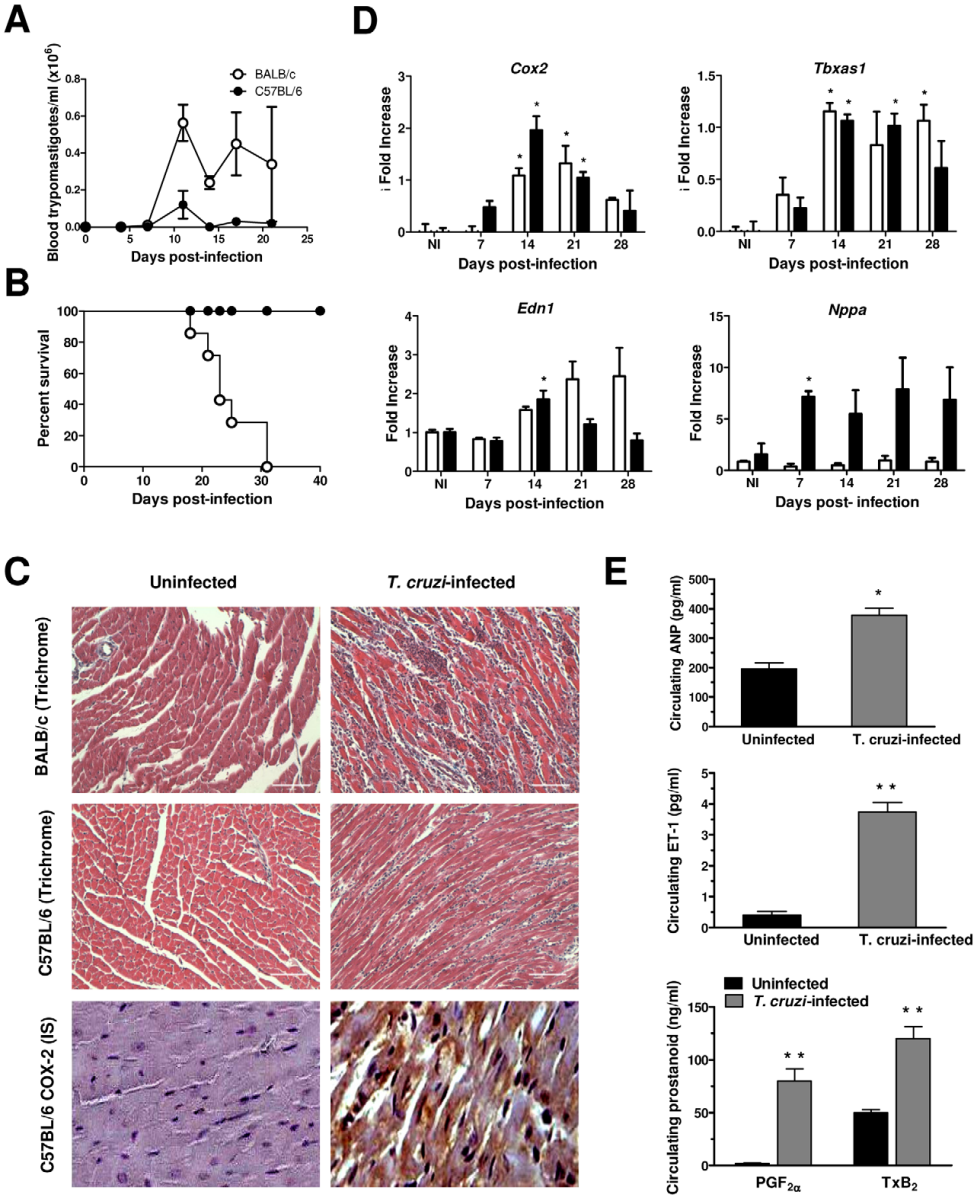
*Trypanosoma cruzi* infection induces *Cox2*, *Tbxas1*, *Edn1* and *Nppa* in infected heart tissue. (A and B) C57BL/6 (*black circles*) and BALB/c (*white circles*) mice were infected with 2×10^3^ blood-trypomastigote forms of the Y strain. (A) Parasitemia expressed as the mean ± standard error of the mean (s.e.m.) of the number of parasites per 5 µl of blood. (B) Percent of mice survival. Results are representative of 2 independent experiments, each performed with 6 mice per group. (C) Tissue inflammation, parasitism and COX-2 expression in heart from uninfected (left panels) and *T. cruzi*-infected (21 days post-infection, right panels) mice. Representative results of histological analysis (Mason's trichrome staining) of cardiac tissue specimens from BALB/c and C57BL/6 mice (top and center panels, respectively) are shown. Bars = 100 µm. Bottom panels display representative results of COX-2 immunostaining (IS) in the hearts from C57BL/6 mice. Original magnification for microphotographs ×400. (D) *Cox2* (COX-2), *Tbxas1* (TXS), *Edn1* (ET-1) and *Nppa* (ANP) gene expression in the heart during the acute phase of infection in C57BL/6 and BALB/c mice. RNA from heart tissue at different days post-infection was used to perform RT-PCR with specific probes, and normalized to ribosomal 18S RNA as described in ‘Materials and Methods’. Values are expressed as means ± s.e.m. from 3 independent infections, each performed with 3 mice per group. *P<0.05. (E) Levels of circulating peptides (ET-1 and ANP) and eicosanoids (PGF_2α_ and TxB_2_) in the sera of uninfected (*black bars*) and *T. cruzi*-infected (*grey bars*) C57BL/6 mice. Mouse sera were collected before and after 21 days of infection, and were assayed in triplicate by capture ELISA for ANP (top panel), ET-1 (central panel), PGF_2α_ and TxB_2_ (bottom panel). Each bar represents the mean values for groups of 6 mice ± s.e.m. Similar results were obtained in two additional experiments. ^*^ *P*<0.05; ^**^ *P*<0.01.

As shown in previous works from our group [30], [37], C57BL/6 mice proved susceptible to infection with the Y strain of *T. cruzi*, albeit less severely than BALB/c mice, and survived acute infection (Figure 1A,B). Intense myocardial parasitism and inflammatory pathology were observed at 21 days of infection, together with enhanced COX-2 expression revealed by immunohistochemistry in both cardiomyocytes and heart-infiltrating leukocytes (Figure 1C). Accordingly, *T. cruzi*-infected C57BL/6 mice showed an augmented (up to 100 fold) expression of myocardial *Cox2* mRNA (Figure 1D) coincident with the highest parasite burden in the heart and maximum severity of myocarditis [30]. In addition, we detected a parallel increase (up to 15 fold) in the expression of the TXS gene (*Tbxas1*). However, no effect was observed on the expression of *Cox1* mRNA (data not shown). Overall, results similar to those above were found in *T. cruzi*-infected BALB/c mice. Moreover, mRNA levels of ET-1 (*Edn1*) and ANP (*Nppa*), a prognostic marker for impairment in cardiac function of chagasic patients [28], were up-regulated in heart tissue of infected C57BL/6 mice (Figure 1D). Upon infection, ET-1 increased in the two mouse genetic backgrounds. This enhanced mRNA expression in the heart of infected animals was accompanied by elevated serum levels of both peptides and circulating eicosanoids (TXB_2_ and PGF_2α_) (Figure 1E). It is important to note that observed values from BALB/c and C57BL/6 animals cannot be directly compared to each other, since data are normalized to non-infected values that can differ between both mouse strains.

### *Trypanosoma cruzi-* and endothelin-1-regulated *Cox2* expression in mouse cardiomyocytes

**Figure 2:**
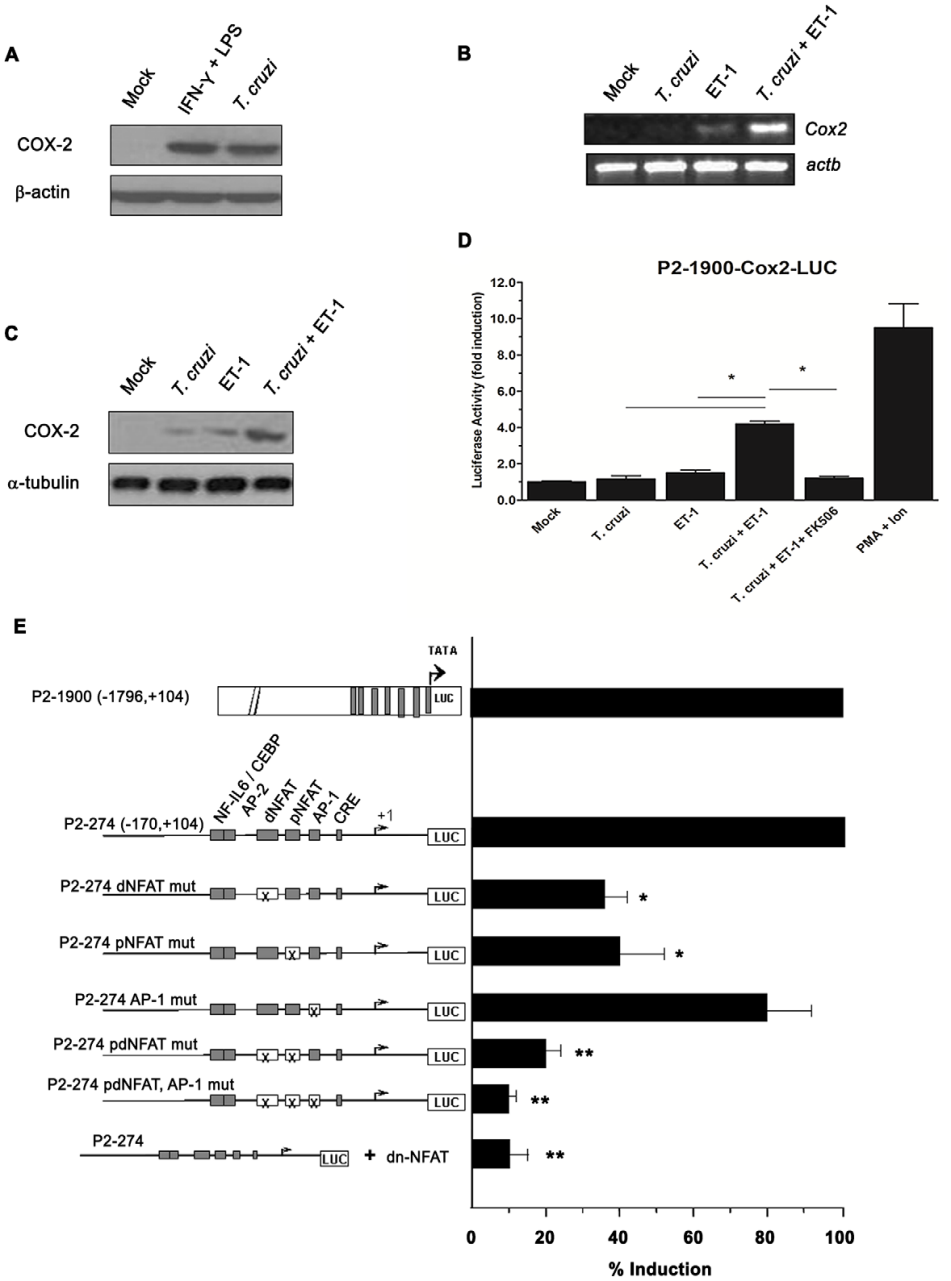
*Trypanosoma cruzi* infection of endothelin-1-pre-treated HL-1 cardiomyocytes induces cyclooxygenase-2 expression. (A) COX-2 protein expression in primary BALB/c cardiac myocytes infected with *T. cruzi*. Neonatal mouse heart cells were isolated and *ex vivo* infected with Y strain trypomastigotes (cell∶parasite ratio 1∶5) for 24 h. To obtain a positive control, the cells were incubated with 25 U/ml recombinant IFN-γ plus 1 µg/ml LPS. Uninfected cells (Mock) were used as controls. The levels of COX-2 and β-actin proteins were analysed by immunoblotting as described under ‘Materials and methods’. (B) Effects of ET-1 pre-treatment and *T. cruzi* infection of HL-1 cardiomyocytes on *Cox2* mRNA expression. HL-1 atrial muscle cells were stimulated with 0.3 nM ET-1 for 2 h, and/or infected with *T. cruzi* trypomastigotes (cell∶parasite ratio 1∶5) for 3 h, and the levels of *Cox2* mRNA were assessed by reverse transcription and PCR; *Actb* (β-actin) was used as a loading marker. (C) Effects of ET-1 pre-treatment and *T. cruzi* infection of HL-1 cardiomyocytes on COX-2 protein expression. HL-1 atrial muscle cells were stimulated with 0.3 nM ET-1 for 2 h, and/or infected with *T. cruzi* trypomastigotes for 3 h, and the levels of COX-2 and α-tubulin proteins were analysed by immunoblotting. (D) Effects of ET-1 pre-treatment and *T. cruzi* infection of HL-1 cardiomyocytes on the inducibility of the *Cox2* promoter. Cells were transiently transfected with the P2-1900-Cox-2-LUC reporter construct, and then stimulated with 0.3 nM ET-1 for 2 h, and/or infected with trypomastigotes for 3 h. For some experiments, FK506 (100 ng/ml) was added to [*T. cruzi*+ET-1]-activated cardiomyocytes. PMA+Ion was used as a standard stimulus. Luciferase activity is expressed as fold induction relative to the transfection with empty expression vector. Data are the means ± s.e.m. of three independent experiments, each performed in triplicate. *P<0.05. (E) Involvement of NFAT in *Cox2* induction by *T. cruzi* plus ET-1. HL-1 cells were transiently transfected with the P2-1900-Cox-2-LUC reporter construct, with the P2-274-Cox-2 promoter construct, or with the same construct containing distal and/or proximal NFAT sites (dNFAT and pNFAT, respectively), and/or actvated protein-1 (AP-1) site mutated (indicated by X). For some experiments, the cells were transiently co-transfected with the P2-274-Cox-2-LUC reporter plasmid along with a dominant-negative version of NFAT (dn-NFAT). Three hours later, the cells were stimulated with ET-1 (0.3 nM) for 2 h and infected with *T. cruzi* parasites for 3 h. Luciferase activity is expressed as percentage of induction (mean ± s.e.m.) relative to that achieved in P2-1900-Cox-2-LUC transfected cells. One out of three separate experiments performed is shown. *P<0.05; ** *P*<0.001 (respect to the P2-274 construct).

The observed *Cox2* mRNA expression in infected heart could come from infected cardiomyocytes, endothelial cells, fibroblasts and/or infiltrating leukocytes. Hence, we tested whether cardiomyocytes up-regulate *Cox2* upon *T. cruzi* infection *in vitro*. A strong induction of COX-2 protein expression was observed in neonatal cardiomyocyte primary cultures infected with *T. cruzi*, comparable to that induced by a well-known pro-inflammatory stimulus as LPS plus IFNγ (Figure 2A). To better examine the molecular regulatory mechanism of gene expression of this inducible enzyme by infection, we used the terminally differentiated murine HL-1 cardiomyocyte cell line infected with *T. cruzi*. Although some reports have described an impaired inflammatory ability of HL-1 cells to express NO synthase-2 or to activate NF-κB [38], others find the opposite [39]. Nonetheless, in our hands these cells retain contractile and phenotypic characteristics of the adult cardiomyocytes and they are much better suitable for transfection experiments than immature cardiac myocytes, as it has been described [40]. After 3 h of parasite infection, *Cox2* mRNA could not be detected. Similarly, a very weak *Cox2* induction was also noted in cardiomyocytes cultured in the presence of 0.3 nM ET-1. However, when ET-1-pre-treated HL-1 cells were infected with *T. cruzi* trypomastigotes ([*T. cruzi*+ET-1]), a strong increase in *Cox2* mRNA expression was detected (Figure 2B). These findings were confirmed by analysing COX-2 protein (Figure 2C).

The above results suggested that the combined effect of *T. cruzi* infection and ET-1 treatment on *Cox2* expression was taking place at the transcriptional level. To confirm this, HL-1 cardiac cells were transfected with a *Cox2* promoter/luciferase construct spanning from nucleotide −1796 to +104 bp relative to the human *Cox2* gene transcription start site (P2-1900-Cox-2-LUC). As shown in Figure 2D, *T*. *cruzi* plus ET-1 (0.3 nM) induced a four-fold increment (*P*<0.05) in luciferase activity in transiently transfected cells compared to untreated controls. In contrast, *T. cruzi*-infected cardiomyocytes and ET-1-stimulated uninfected cells showed very little increase. Interestingly, addition of the Cn inhibitor FK506 (100 ng/ml) significantly attenuated [*T. cruzi*+ET-1]-mediated induction of *Cox2* promoter.

### Transcriptional regulation of the *Co*x*2* promoter by the combined effect of *Trypanosoma cruzi* and endothelin-1

To map the *Cox2* promoter region responsible for [*T. cruzi*+ET-1] inducibility, we used several *Cox2* promoter deletion/mutation constructs. Deletion up to −170 (P2-1900 to P2-274) of the *Cox2* promoter region did not significantly affect [*T. cruzi*+ET-1] inducibility (Figure 2E). Given the relevance of the region spanning from nucleotides −170 to −46 for the recorded induction of the *Cox2* promoter, we next determined the contribution of the known transcription factor sites present in this region [19] to the overall transcriptional regulation of [*T. cruzi*+ET-1]-dependent *Cox2* expression. Transfection experiments showed that mutation of the dNFAT (P2-274 dNFAT mut) or pNFAT (P2-274 pNFAT mut) sites resulted in a 65 and a 60% loss in the [*T. cruzi*+ET-1]-induced *Cox2* promoter activity, respectively, whereas double mutation of both NFAT (P2-274 p- and dNFAT mut) sites drastically reduced this activation. Conversely, mutagenesis of the AP-1-like site (P2-274 AP-1 mut) present in this region did not significantly diminish the inducibility of the *Cox2* promoter by [*T. cruzi*+ET-1]. To further confirm the central role of NFAT activation in the transcriptional regulation mediated by *T. cruzi* in ET-1-stimulated HL-1 cells, we co-transfected a dominant-negative version of NFAT (dnNFAT), previously described to abolish NFAT-dependent promoter activity [19], together with the P2-274-Cox-2-LUC plasmid. Interestingly, expression of dnNFAT abrogated [*T. cruzi*+ET-1]-induced transcription of the reporter (Figure 2E), supporting the hypothesis of the involvement of NFAT signaling in the regulation of *Cox2* gene expression by the cooperation between ET-1 and *T. cruzi* infection in cardiomyocytes.

### *Trypanosoma cruzi* infection leads to activation of the Ca^2+^/Calcineurin/NFAT intracellular signaling pathway in endothelin-1-treated cardiomyocytes

**Figure 3:**
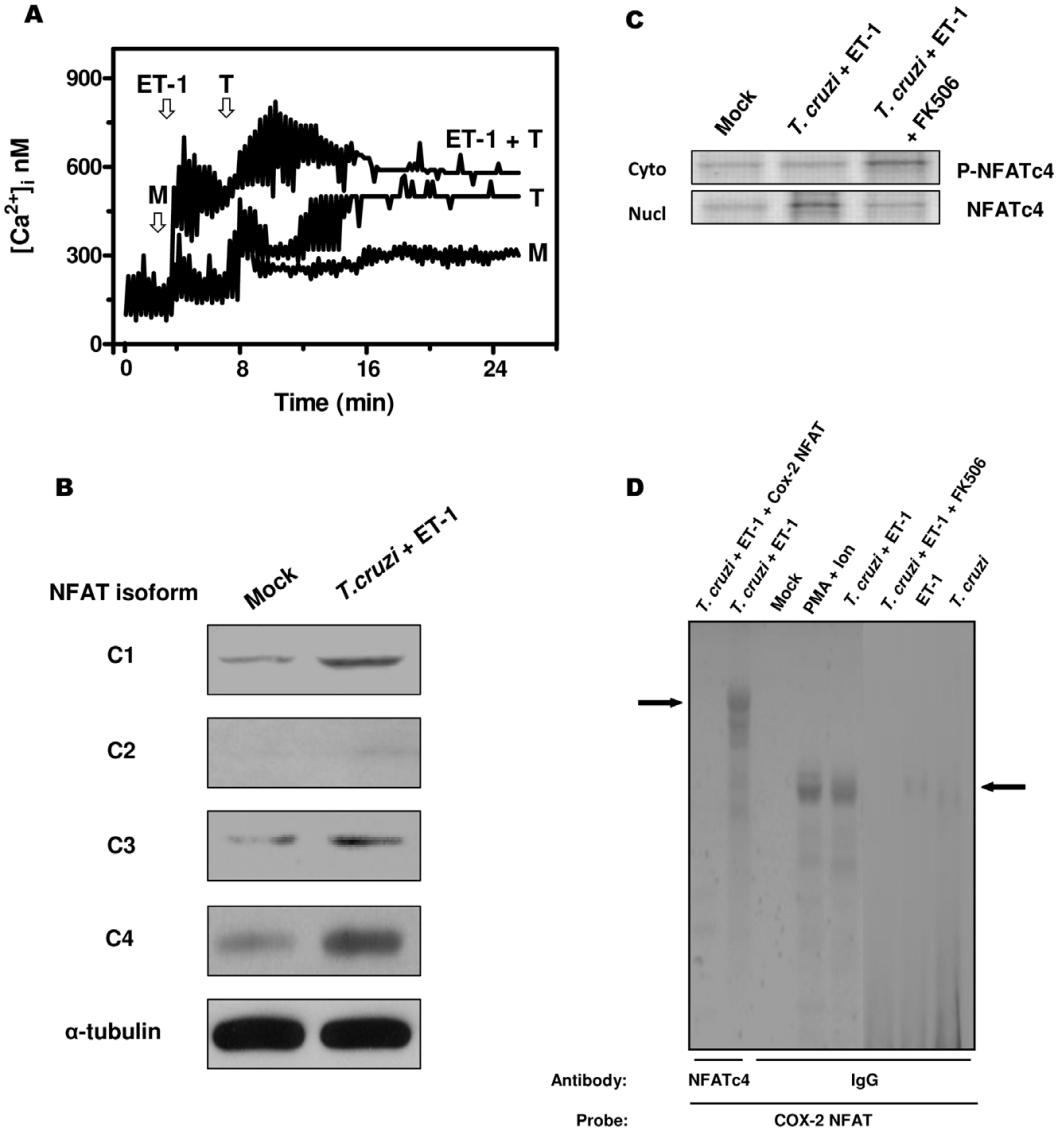
Activation of the Ca^2+^/Calcineurin/NFAT intracellular signaling pathway in endothelin-1-stimulated and *Trypanosoma cruzi*-infected cardiomyocytes. (A) HL-1 cells, exposed or not to 0.3 nM ET-1, were loaded with the Ca^2+^ indicator Fura-2/M and changes in [Ca^2+^]_i_ upon *T. cruzi* infection were recorded. Uninfected cells were used as a control. Arrows indicate the time (min) when either culture medium (M) or *T. cruzi* trypomastigotes (T) was added. The results presented are representative of three independent experiments. (B) ET-1 stimulated and *T. cruzi*-infected HL-1 cardiomyocytes were disrupted and the protein expression of the four NFAT isoforms (c1 to c4) was analysed by immunoblotting. Alpha-tubulin protein levels were determined as a control of loading. (C) HL-1 cells were incubated for 2 h with ET-1 (0.3 nM) and subsequently infected with *T. cruzi* trypomastigotes for 3 h. For some experiments, FK506 (100 ng/ml) was added 1 h before stimulation. Fractionated extracts from both untreated and treated cells were analysed by immunoblotting with an antiserum to NFATc4. The phosphorylated cytosolic (P-NFATc4) or dephosphorylated nuclear (NFATc4) forms of the factor are indicated. Cyto, cytosolic extracts; Nucl, nuclear extracts. (D) Electrophoretic mobility shift assay (EMSA) analysis to determine NFATc4 binding to the NFAT sites of the *Cox2* gene (Cox-2 NFAT). HL-1 myocytes were stimulated with 0.3 nM ET-1 for 2 h and/or infected with *T. cruzi* trypomastigotes for 3 h. For some experiments, FK506 (100 ng/ml) was added 1 h before stimulation. Mock-treated cells were considered as controls. PMA (15 ng/ml) supplemented with 1 µM Ion was used as a standard stimulus. Nuclear extracts were analysed by EMSA using a Cox-2 NFAT radiolabeled probe. A 50-fold molar excess of unlabeled Cox-2 NFAT oligonucleotide (*T. cruzi*+ET-1+Cox-2 NFAT) was added to determine specific binding. NFATc4 antibody or normal rabbit IgG was added to the extracts before incubation with the probe. Arrows indicate specific supershifted complexes. This is representative of at least three independent experiments.

*T. cruzi* trypomastigote invasion of cardiac myocytes triggers a transient [Ca^2+^]_i_ elevation [41]. Similarly, upon the addition of trypomastigotes to HL-1 cells, we observed a transient [Ca^2+^]_i_ response associated to a considerable, sustained increase in [Ca^2+^]_i_ during the invasion process (Figure 3A). Comparable outcome, although with higher [Ca^2+^]_i_ levels, was obtained in *T. cruzi*-infected HL-1 cells pre-treated with 0.3 nM ET-1.

In HL-1 cells, basal expression of several isoforms of NFAT proteins (c1, c3 and c4) was detected by immunoblot analysis. Interestingly, stimulation with *T. cruzi* plus ET-1 induced a remarkable increase in the expression of NFATc4 and to a lesser extent, NFATc1 and NFATc3 (Figure 3B). Moreover, NFATc4 was present in the cytoplasm of untreated cardiac cells, but upon parasite infection of ET-1-stimulated cardiomyocytes, it was translocated into the nucleus. Pre-treatment with FK506 (100 ng/ml), a Cn inhibitor, prevented this translocation, thereby resulting in an accumulation of cytoplasmic NFATc4 protein (Figure 3C). To a much lesser extent, we also observed NFATc1 and NFATc3 migration to the nucleus (data not shown). Together, the above results indicate the activation of the NFATc4 isoform by [*T. cruzi*+ET-1] through a Ca^2+^/Cn signaling process.

To analyse NFATc4 binding to the NFAT sequences of the *Cox2* promoter, we performed EMSAs with nuclear extracts of atrial HL-1 myocytes (Figure 3D). PMA (15 ng/ml) supplemented with Ion (1 µM) was used as a control stimulus. The NFAT oligonucleotide probe from *Cox2* promoter specifically bound nuclear proteins from [*T. cruzi*+ET-1]- and [PMA+Ion]-treated HL-1 cells, which was efficiently competed with a 50-fold molar excess of cold oligonucleotide (Cox-2-NFAT). These inducible complexes were severely diminished in nuclear extracts from cells stimulated with *T. cruzi* plus ET-1 in the presence of FK506. No NFAT binding could be demonstrated in response to ET-1 stimulation in the absence of parasites or *T. cruzi* infection alone. To determine unambiguously the presence of the NFATc4 protein in the complexes, we performed super shifting with an NFATc4-specific antibody. This antibody clearly displaced the migration of the bound probe, allowing the formation of more retarded complexes likely constituted by DNA/NFAT/antibody (Figure 3D). As the NFATc4-specific antibody completely supershifted the complex, it is indicative that c4, but no other NFAT isoform, is bound to *Cox2* promoter DNA in detectable amount. As a negative control, normal rabbit IgG was used. Taken together, these data suggest the binding of NFATc4 to the corresponding sites within the *Cox2* promoter in response to *T. cruzi* infection of ET-1-pre-treated HL-1 cells.

### *Trypanosoma cruzi* infection of endothelin-1-treated HL-1 cardiomyocytes enhances the production of eicosanoids and atrial natriuretic peptide

**Figure 4:**
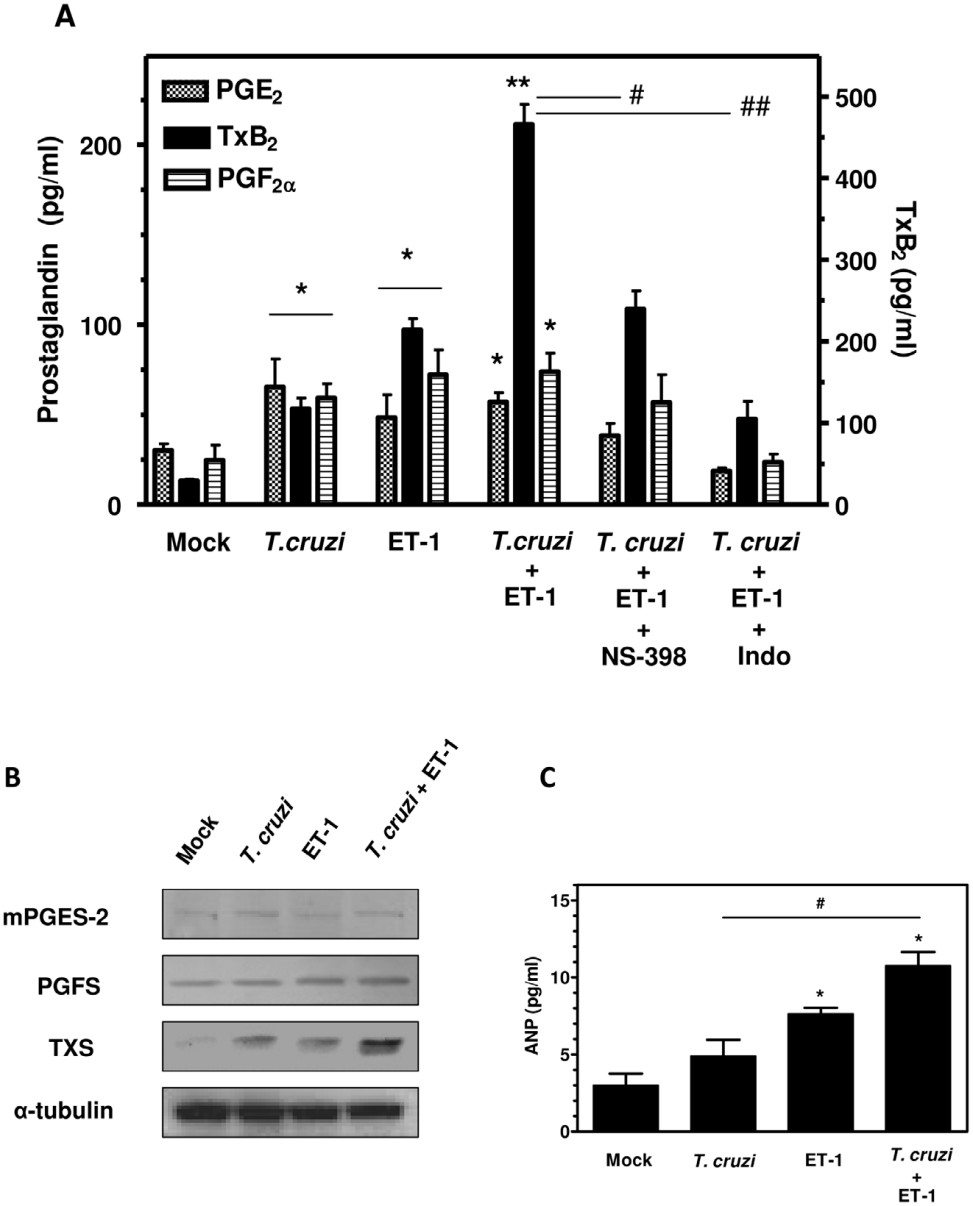
Production of eicosanoids and atrial natriuretic peptide by endothelin-1-stimulated and *Trypanosoma cruzi*-infected HL-1 cardiac cells. (A) Cardiomyocytes were serum-starved for 12 h, then incubated for 1 h in the presence of cyclooxygenase inhibitors (10 µM indomethacin -Indo- or 10 µM NS-398) and further stimulated with 0.3 nM ET-1 for 2 h. After treatment, the cells were infected with *T. cruzi* trypomastigotes for 24 h. HL-1 myocytes infected with the parasite or stimulated with ET-1 alone were included in the assay. Culture supernatants were collected and analysed for PGE_2_, PGF_2α_ and TxB_2_ (TxA_2_ stable metabolite) by ELISA (Cayman). The results represent means ± s.e.m. of three individual experiments assayed in triplicate. *P<0.05 and **P<0.001 compared with mock-treated cells; ^#^ *P*<0.05 and ^##^ *P*<0.001 compared with NS-398- and Indo-treated cells, respectively. (B) Effects of *T. cruzi* infection and ET-1 stimulation on the expression of prostanoid terminal synthases in HL-1 cardiac cells. Myocytes were incubated with 0.3 nM ET-1 for 2 h and/or infected with *T. cruzi* trypomastigotes for 3 h. Uninfected and mock-treated cells were used as controls. Immunoblot analysis of the protein expression of prostanoid terminal synthases (mPGES-2, PGFS and TXS) after exposure to stimulus and/or parasite is shown. Alpha-tubulin was used as loading control. The results presented are representative of three independent experiments with similar outcome. (C) HL-1 cells were treated with ET-1 (0.3 nM) for 2 h and/or *T. cruzi*-infected for 24 h and supernatants were collected. Uninfected and mock-treated cells were used as controls. ANP release (mean ± s.e.m.) was analyzed using an ELISA kit (Kamiya Biomedical) following the manufacturer's instructions. The results are representative of three independent experiments performed in quadruplicate. Statistically significant differences are indicated (*P<0.05, compared with mock; ^#^ *P*<0.05, [*T. cruzi*+ET-1]-activated cells *versus T. cruzi*-infected myocytes).

To assess whether [*T. cruzi*+ET-1]-mediated induction of *Cox2* expression was associated with an increase in its enzymatic activity, eicosanoid release by HL-1 cells was measured. Compared to mock-treated cells, stimulation of myocytes with 0.3 nM ET-1, or trypomastigote infection over a 24-h period, or the combination of both, induced a significant production of COX metabolites, mainly TXB_2_, the stable metabolite of TXA_2_, and prostaglandins E_2_ (PGE_2_) and PGF_2α_. Particularly, a striking increase of TXB_2_ levels, significantly higher than those obtained with *T. cruzi* and ET-1 separately, was detected in response to [*T. cruzi*+ET-1] (Figure 4A). Likewise, induction of the Ca^2+^/Cn/NFAT/COX-2 pathway and eicosanoid production were also achieved in ET-1-primed HL-1 cells exposed to a parasite lysate preparation, thereby suggesting that cardiac cell invasion by trypomastigotes is not absolutely required to produce the cooperative effect with the peptide (not shown). TXB_2_, PGE_2_ and PGF_2α_ synthesis was drastically reduced in the cells incubated with indomethacin (10 µM), a non-steroidal anti-inflammatory drug known to inhibit both COX-1 and COX-2 enzymatic activity, or with a COX-2-selective inhibitor (NS398, 10 µM), indicating the important involvement of COX-2 in eicosanoid production upon ET-1 stimulation and *T. cruzi* infection of HL-1 cardiomyocytes. Treatment of HL-1 cells with COX inhibitors or Cn antagonist had no significant effect on cardiomyocyte-*T. cruzi* association and did not affect the capacity of the parasites to transform into amastigotes and multiply intracellularly (not shown). Furthermore, analyses for microsomal prostaglandin E synthase-2 (mPGES-2), prostaglandin F synthase (PGFS) and thromboxane synthase (TXS), enzymes that convert the COX product PGH_2_ to PGE_2_, PGF_2α_ and TXA_2_, respectively, revealed that [*T. cruzi*+ET-1] also induced the expression of TXS and PGFS proteins in atrial HL-1 myocytes (Figure 4B).

In addition, stimulation with ET-1 promoted a three-fold increased (*P*<0.05) release of ANP. Compared to that observed in mock-treated controls, *T. cruzi* also up-regulated ANP levels in the supernatants of 24-h-infected cells, which were significantly augmented by the cooperative action of [*T. cruzi*+ET-1] (Figure 4C).

## Discussion

*Trypanosoma cruzi* induces multiple responses in the heart, a critical organ of infection and pathology in the host. We herein demonstrated that *Cox2* mRNA and protein are induced in mouse heart tissue during *T. cruzi* infection correlating with cardiac parasite load and myocarditis. This up-regulation was also associated to induction of TXS and of two markers of heart dysfunction previously implicated in Chagas' disease pathogenesis, such as ET-1 and ANP [7], [10], [27]. Up-regulation of *Cox2* mRNA and protein in myocardial tissue of infected C57BL/6 mice is consistent with a previous report [22] that revealed increased COX-2 protein expression in the heart of infected BALB/c mice. Moreover, several evidences have suggested a role of cyclooxoygenase-derived eicosanoids in the cardiopathogenesis of Chagas' disease (revised in [42], [43]).

Using adult HL-1 atrial myocytes, we further demonstrated that cooperation between *T. cruzi* and ET-1 stimulated *Cox2* mRNA and protein expression leading to the release of eicosanoids. ET-1 seems to be mainly implicated in the establishment of chagasic cardiomyopathy rather than in the control of infection. Previous studies on *T. cruzi*-infected ET-1 null mice have highlighted the pathogenic role of cardiac myocyte-derived ET-1 in Chagas' heart disease, but these animals did not display higher parasitemia nor lower survival rate than infected wild-type mice [8]. In chagasic heart dysfunction, locally produced ET-1 acts on cardiac myocytes in both an autocrine and/or paracrine manner and chronically induces muscle injury [5], [7]. In addition, exposure of neonatal rat ventricular cardiomyocytes to ET-1 has been shown to result in higher COX-2 and prostacyclin formation [21], [44]. In our study, ET-1 induced a dose-dependent increase (not shown) in COX-2 activity and eicosanoid biosynthesis in HL-1 cells subsequently infected with *T. cruzi*. To mimic the pathological microenvironment characteristic of *T. cruzi*-mediated cardiomyopathy, a 0.3 nM ET-1 concentration, close to that detected in the circulation of infected mice and patients exhibiting cardiac involvement [5], [6], was selected for pre-treatment of cardiomyocytes.

*Trypanosoma cruzi* invasion of HL-1 cells increased [Ca^2+^]_i_, similar to previous report on primary cardiomyocytes [41]. Furthermore, ET-1 induces Ca^2+^ release in cardiac myofibers [45]. Alterations in [Ca^2+^]_i_ regulation are frequently recorded in Chagas' disease. In cardiomyocytes from chagasic patients there is a dysregulation of the diastolic [Ca^2+^]_i_, while Ca^2+^ channel blockers display therapeutic potential against chronic chagasic cardiomyopathy [46], [47]. It has been largely established the requirement for sustained increases, including Ca^2+^ oscillation frequency, in [Ca^2+^]_i_ to mediate Cn activation and the nuclear translocation of NFAT [48]. Few studies so far have addressed the impact of *T. cruzi* infection on the Cn/NFAT pathway in host cells. NFAT has been identified as an important element in innate immunity to *T. cruzi* and also involved in parasite immune evasion [49], [50]. The Ca^2+^/Cn/NFAT pathway has proven functional in adult mouse heart muscle cells and ET-1 has been shown to activate this signaling route in HL-1 atrial myocytes [51], [52]. Noticeably, NFAT proteins have been described as key molecules for the regulation of *Cox2* gene transcription in many different cell types [19], [53]–[55]. Our present report constitutes the first demonstration that the cooperative effect of ET-1 and *T. cruzi* infection transcriptionally controls *Cox2* expression through activation of the Cn/NFATc4 signaling cascade in cardiomyocytes. Particularly, the two NFAT binding sites in the *Cox2* promoter appear to be critical for the observed induction. Mutation of any of these sites strongly diminished *Cox2* transcription raised by *T. cruzi* infection of ET-1-stimulated cardiomyocytes, and dominant negative NFAT prevented that stimulation.

Interestingly, this Cn/NFAT pathway has a pivotal role in pathological cardiac hypertrophy [26]. In this regard, we found that ET-1 plus *T. cruzi* infection leads to enhanced production of the pro-hypertrophic ANP, a prognostic factor for impairment in cardiac function of chagasic patients [28]. Augmented ANP was previously observed in atrial muscle cells upon ET-1 stimulation [56] and, during *T. cruzi* infection, ET-1 and ANP seem to be important late factors in myocardial remodeling and hypertrophy [10], [27]. Increased ANP production is somehow linked to the myocardial regulatory pathway induced by [*T. cruzi*+ET-1]. Thus, PGE_2_ and PGF_2α_ are known to promote ANP synthesis and release [57], [58], while Ca^2+^ influx is involved in ET-1-triggered ANP expression [59]. More interestingly, NFATc4 was found to regulate several hypertrophy-associated gene transcription in cardiomyocytes, including ANP [26], [58]. Taken the data together, it is likely that Ca^2+^ elevation, induced by [*T. cruzi*+ET-1], has led to NFATc4 activation, COX-2 induction and augmented ANP secretion by HL-1 cells.

A dual role of cyclooxygenase-derived eicosanoids in the course of Chagas' disease has been postulated (revised in [42], [43]). Morever, the same COX metabolites that mediate host survival during the acute phase may contribute to the progression of cardiac remodeling and heart damage in the chronic phase [60]. The mechanisms involved in the increased prostanoid production in parasite-infected hosts are not yet fully understood. Our findings indicate that the combined effect of ET-1 priming and *T. cruzi* infection mimics what likely takes place in the heart during infection, inducing eicosanoid-forming enzyme activity through the Ca^2+^/Cn/NFAT signaling pathway, and leading to enhanced release of prostanoids by atrial cardiomyocytes. Acutely infected mice display elevated PGF_2α_ plasma levels, whereas PGE_2_ has been found to favor the development of cardiac fibrosis and functional deficits after infection by *T. cruzi* [23], [61]. TXA_2_, measured as the stable metabolite TXB_2_, is the main eicosanoid produced during chronic infection with *T. cruzi* and this pro-inflammatory agent could be responsible of several of the pathophysiological features of chagasic cardiomyopathy [23], [24]. TXA_2_ may exacerbate cardiomyocyte apoptosis, facilitate cytokine biosynthesis by monocytes, activate endothelial cells, and also promote platelet activation, aggregation and degranulation [62]. It is conceivable that the liberated TXA_2_ might play a role in a feedback loop for ET-1 expression/response, as efficient regulation of ET-1 by a TXA_2_ mimetic in rat heart smooth muscle cells has been documented [63]. Moreover, the released PGF_2α_ could further induce COX-2 expression and activity, as occurs in carcinoma cells [64]. Enhanced levels of eicosanoids synthesized by [*T. cruzi*+ET-1]-activated HL-1 cells were down-regulated by addition of COX-2 inhibitors, indomethacin or NS398. In this regard, meloxicam or etoricoxib, two specific COX-2 inhibitors, minimized the amount of inflammation and fibrosis in the cardiac tissue of infected mice, whereas delayed treatment with aspirin, which blocks COX-1 and COX-2 indistinctly, improved cardiac dysfunction in a murine model of Chagas' heart disease [22], [60]. However, the potential benefits of COX inhibition for chronic chagasic patients are still unknown. Even though *T. cruzi*-derived TXA_2_ and PGF_2α_ have been associated with pathogenesis [24], [43], no consistent evidence of parasite COX-2 and TXAS expression is available so far. As we detected overexpression of myocardial enzymes by using mouse-specific probes/antibodies and dampened eicosanoid production in cardiomyocytes treated with mammalian enzyme-specific inhibitors, our data mostly reflect the contribution of prostanoids secreted by host cells to Chagas' myocarditis.

In conclusion, we have demonstrated that eicosanoid-converting enzymes are expressed in the infected heart and also that cardiomyocytes respond to ET-1 and *T. cruzi* infection by induction of COX-2 through activation of the Ca^2+^/Cn/NFAT intracellular signaling pathway. The cooperation between *T. cruzi* and ET-1 also led to overproduction of eicosanoids and the pro-hypertrophic factor ANP. These results support an important role for NFAT in [*T. cruzi*+ET-1]-dependent induction of key agents of pathogenesis in chronic chagasic cardiomyopathy. Identification of the Ca^2+^/Cn/NFAT cascade as mediator of cardiovascular pathology in Chagas' disease advances our understanding of host-parasite relationship and may help define novel potential targets for therapeutic interventions to ameliorate or prevent cardiomyopathy during chronic *T. cruzi* infection.

## References

[1] PAHO 2007: Pan American Health Organization 2007 (2007) Meeting Conclusions and Recommendations from the Joint IPA-AMCHA Annual Meeting (Quito, Ecuador); Technical Guidelines for Prevention and Control of Chagas Disease; PAHO/MSF Regional Consultation on the Organization and Structure of Health Care (IEC) on Congenital Chagas Disease (CLAP, Montevideo, 17–18 May 2007).

[2] Rocha MO, Teixeira MM, Ribeiro AL (2007) An update on the management of Chagas cardiomyopathy. Exper Rev Anti Infect Ther 5: 727–743. 10.1586/14787210.5.4.727 17678433

[3] Tanowitz HB, Kaul DK, Chen B, Morris SA, Factor SM, et al (1996) Compromised microcirculation in acute murine *Trypanosoma cruzi* infection. J Parasitol 82: 124–130. 8627481

[4] Mukherjee S, Huang H, Weiss LM, Costa S, Scharfstein J, et al (2003) Role of vasoactive mediators in the pathogenesis of Chagas' disease. Front Biosci 8: e410–419. 12700081 10.2741/1103

[5] Petkova SB, Tanowitz HB, Magazine HI, Factor SM, Chan J, et al (2000) Myocardial expression of endothelin-1 in murine *Trypanosoma cruzi* infection. Cardiovasc Pathol 9: 257–265. 11064272 10.1016/s1054-8807(00)00045-4

[6] Salomone OA, Caeiro TF, Madoery RJ, Amuchástegui M, Omelinauk M, et al (2001) High plasma immunoreactive endothelin levels in patients with Chagas' cardiomyopathy. Am J Cardiol 87: 1217–1220. 11356406 10.1016/s0002-9149(01)01502-8

[7] Petkova SB, Huang H, Factor SM, Bouzahzah B, Pestell RG, et al (2001) The role of endothelin in the pathogenesis of Chagas' disease. Int J Parasitol 31: 499–511. 11334935 10.1016/s0020-7519(01)00168-0

[8] Huang H, Yanagisawa M, Kisanuki YY, Jelicks LA, Chandra M, et al (2002) Role of cardiac myocyte-derived endothelin-1 in chagasic cardiomyopathy: molecular genetic evidence. Clin Sci (Lond) 103, Suppl 48: 263S–266S. 12193100 10.1042/CS103S263S

[9] Tanowitz HB, Wittner M, Morris SA, Zhao W, Weiss LM, et al (1999) The putative mechanistic basis for the modulatory role of endothelin-1 in the altered vascular tone induced by *Trypanosoma cruzi* . Endothelium 6: 217–230. 10365773 10.3109/10623329909053412

[10] Tanowitz HB, Huang H, Jelicks LA, Chandra M, Loredo ML, et al (2005) Role of endothelin 1 in the pathogenesis of chronic chagasic heart disease. Infect Immun 73: 2496–2503. 15784596 10.1128/IAI.73.4.2496-2503.2005 PMC1087455

[11] Mulder P, Richard V, Derumeaux G, Hoggie M, Henry JP, et al (1997) Role of endogenous endothelin in chronic heart failure: effect of long-term treatment with an endothelin antagonist on survival, hemodynamics, and cardiac remodeling. Circulation 96: 1976–1982. 9323089 10.1161/01.cir.96.6.1976

[12] Morris SA, Hatcher V, Bilezikian JP, Tanowitz HB, Wittner M (1988) Alterations in intracellular calcium following infection of human endothelial cells with *Trypanosoma cruzi* . Mol Biochem Parasitol 29: 213–221. 3045542 10.1016/0166-6851(88)90076-x

[13] Hassan GS, Mukherjee S, Nagajyothi F, Weiss LM, Petkova SB, et al (2006) *Trypanosoma cruzi* induces proliferation of vascular smooth muscle cells. Infect Immun 74: 152–159. 16368968 10.1128/IAI.74.1.152-159.2006 PMC1346667

[14] Kawamura T, Ono K, Morimoto T, Akao M, Iwai-Kanai E, et al (2004) Endothelin-1-dependent nuclear factor of activated T lymphocyte signaling associates with transcriptional coactivator p300 in the activation of the B cell leukemia-2 promoter in cardiac myocytes. Circ Res 94: 1492–1499. 15117818 10.1161/01.RES.0000129701.14494.52

[15] Zhu W, Zou Y, Shiojima I, Kudoh S, Aikawa R, et al (2000) Ca^2+^/calmodulin-dependent kinase II and calcineurin play critical roles in endothelin-1-induced cardiomyocyte hypertrophy. J Biol Chem 275: 15239–15245. 10809760 10.1074/jbc.275.20.15239

[16] Vihma H, Pruunsild P, Timmusk T (2008) Alternative splicing and expression of human and mouse NFAT genes. Genomics 92: 279–291. 18675896 10.1016/j.ygeno.2008.06.011 PMC2577130

[17] Hogan PG, Chen L, Nardone J, Rao A (2003) Transcriptional regulation by calcium, calcineurin, and NFAT. Genes Dev 17: 2205–2232. 12975316 10.1101/gad.1102703

[18] Abdullah HI, Pedraza PL, Hao S, Rodland KD, McGiff JC, et al (2006) NFAT regulates calcium-sensing receptor-mediated TNF production. Am J Physiol Renal Physiol 290: F1110–F1117. 16380462 10.1152/ajprenal.00223.2005

[19] Iñiguez MA, Martínez-Martínez S, Punzón C, Redondo JM, Fresno M (2000) An essential role of the nuclear factor of activated T cells in the regulation of the expression of the cyclooxygenase-2 gene in human T lymphocytes. J Biol Chem 275: 23627–23635. 10816557 10.1074/jbc.M001381200

[20] Kataoka A, Tozaki-Saitoh H, Koga Y, Tsuda M, Inoue K (2009) Activation of P2X7 receptors induces CCL3 production in microglial cells through transcription factor NFAT. J Neurochem 108: 115–125. 19014371 10.1111/j.1471-4159.2008.05744.x

[21] Rebsamen MC, Capoccia R, Vallotton MB, Lang U (2003) Role of cyclooxygenase 2, p38 and p42/44 MAPK in the secretion of prostacyclin induced by epidermal growth factor, endothelin-1 and angiotensin II in rat ventricular cardiomyocytes. J Mol Cell Cardiol 35: 81–89. 12623302 10.1016/s0022-2828(02)00281-x

[22] Abdalla GK, Faria GEL, Silva KT, Castro ECC, Reis MA, et al (2008) *Trypanosoma cruzi*: The role of PGE2 in immune response during the acute phase of experimental infection. Exp Parasitol 118: 514–521. 18163990 10.1016/j.exppara.2007.11.003

[23] Cardoni RL, Antúnez MI (2004) Circulating levels of cyclooxygenase metabolites in experimental *Trypanosoma cruzi* infections. Mediators Inflamm 13: 235–240. 15545053 10.1080/09637480400003022 PMC1781569

[24] Ashton AW, Mukherjee S, Nagajyothi FN, Huang H, Braunstein VL, et al (2007) Thromboxane A2 is a key regulator of pathogenesis during *Trypanosoma cruzi* infection. J Exp Med 204: 929–940. 17420269 10.1084/jem.20062432 PMC2118547

[25] Iwai-Kanai E, Hasegawa K (2004) Intracellular signaling pathways for norepinephrine- and endothelin-1-mediated regulation of myocardial cell apoptosis. Mol Cell Biochem 259: 163–168. 15124920 10.1023/b:mcbi.0000021368.80389.b9

[26] Liu CJ, Cheng YC, Lee KW, Hsu HH, Chu CH, et al (2008) Lipopolysaccharide induces cellular hypertrophy through calcineurin/NFAT-3 signaling pathway in H9c2 myocardiac cells. Mol Cell Biochem 313: 167–178. 18398669 10.1007/s11010-008-9754-0

[27] Benvenuti LA, Aiello VD, Palomino SA, Higuchi M de L (2003) Ventricular expression of atrial natriuretic peptide in chronic chagasic cardiomyopathy is not induced by myocarditis. Int J Cardiol 88: 57–61. 12659985 10.1016/s0167-5273(02)00363-7

[28] Moreira Mda C, Wang Y, Heringer-Walther S, Wessel N, Walther T (2009) Prognostic value of natriuretic peptides in Chagas' disease: a head-to-head comparison of the 3 natriuretic peptides. Congest Heart Fail 15: 75–81. 19379453 10.1111/j.1751-7133.2009.00051.x

[29] Claycomb WC, Lanson NA Jr, Stallworth BS, Egeland DB, Delcarpio JB, et al (1998) HL-1 cells: a cardiac muscle cell line that contracts and retains phenotypic characteristics of the adult cardiomyocyte. Proc Natl Acad Sci U S A 95: 2979–2984. 9501201 10.1073/pnas.95.6.2979 PMC19680

[30] Cuervo H, Pineda MA, Aoki MP, Gea S, Fresno M, et al (2008) Inducible nitric oxide synthase and arginase expression in heart tissue during acute *Trypanosoma cruzi* infection in mice: arginase I is expressed in infiltrating CD68+ macrophages. J Infect Dis 197: 1772–1782. 18473687 10.1086/529527

[31] Cuervo H, Guerrero NA, Carbajosa S, Beschin A, De Baetselier P, et al (2011) Myeloid-derived suppressor cells infiltrate the heart in acute *Trypanosoma cruzi* infection. J Immunol 187: 2656–2665. 21804013 10.4049/jimmunol.1002928

[32] Zhang D, Li J, Wu K, Ouyang W, Ding J, et al (2007) JNK1, but not JNK2, is required for COX-2 induction by nickel compounds. Carcinogenesis 28: 883–891. 17065197 10.1093/carcin/bgl186

[33] Cutrullis RA, Postan M, Petray PB, Corral RS (2009) Timing of expression of inflammatory mediators in skeletal muscles from mice acutely infected with the RA strain of *Trypanosoma cruzi* . Pathobiology 76: 170–180. 19571606 10.1159/000218333

[34] Northrop JP, Ho SN, Chen L, Thomas DJ, Timmerman LA, et al (1994) NF-AT components define a family of transcription factors targeted in T-cell activation. Nature 369: 497–502. 8202141 10.1038/369497a0

[35] Hellmich MR, Ives KL, Udupi V, Soloff MS, Greeley GH Jr, et al (1999) Multiple protein kinase pathways are involved in gastrin-releasing peptide receptor-regulated secretion. J Biol Chem 274: 23901–23909. 10446156 10.1074/jbc.274.34.23901

[36] Martínez-Martínez S, Gomez del Arco P, Armesilla AL, Aramburu J, Luo C, et al (1997) Blockade of T-cell activation by dithiocarbamates involves novel mechanisms of inhibition of nuclear factor of activated T cells. Mol Cell Biol 17: 6437–6447. 9343406 10.1128/mcb.17.11.6437 PMC232496

[37] Calderón J, Maganto-Garcia E, Punzón C, Carrión J, Terhorst C, et al (2012) The receptor Slamf1 on the surface of myeloid lineage cells controls susceptibility to infection by *Trypanosoma cruzi* . PLoS Pathog 8: e1002799. 22807679 10.1371/journal.ppat.1002799 PMC3395606

[38] Cuenca J, Goren N, Prieto P, Martín-Sanz P, Boscá L (2007) Selective impairment of nuclear factor-kappaB-dependent gene transcription in adult cardiomyocytes: relevance for the regulation of the inflammatory response in the heart. Am J Pathol 171: 820–828. 17675583 10.2353/ajpath.2007.061076 PMC1959507

[39] Boltzen U, Eisenreich A, Antoniak S, Weithaeuser A, Fechner H, et al (2012) Alternatively spliced tissue factor and full-length tissue factor protect cardiomyocytes against TNF-α-induced apoptosis. J Mol Cell Cardiol 52: 1056–1065. 22326437 10.1016/j.yjmcc.2012.01.015 PMC3994711

[40] White SM, Constantin PE, Claycomb WC (2004) Cardiac physiology at the cellular level: use of cultured HL-1 cardiomyocytes for studies of cardiac muscle cell structure and function. Am J Physiol Heart Circ Physiol 286: H823–H829. 14766671 10.1152/ajpheart.00986.2003

[41] Garzoni LR, Masuda MO, Capella MM, Gil Lopes A, Leal de Meirelles MNS (2003) Characterization of [Ca^2+^]_i_ responses in primary cultures of mouse cardiomyocytes induced by *Trypanosoma cruzi* trypomastigotes. Mem Inst Oswaldo Cruz 98: 487–493. 12937759 10.1590/s0074-02762003000400010

[42] Maya JD, Orellana M, Ferreira J, Kemmerling U, López-Muñoz R, et al (2010) Chagas disease: Present status of pathogenic mechanisms and chemotherapy. Biol Res 43: 323–331. 21249304

[43] Machado FS, Mukherjee S, Weiss LM, Tanowitz HB, Ashton AW (2011) Bioactive lipids in *Trypanosoma cruzi* infection. Adv Parasitol 76: 1–31. 21884885 10.1016/B978-0-12-385895-5.00001-3 PMC3564251

[44] Manque PA, Probst C, Pereira MC, Rampazzo RC, Ozaki LS, et al (2011) *Trypanosoma cruzi* infection induces a global host cell response in cardiomyocytes. Infect Immun 79: 1855–1862. 21343357 10.1128/IAI.00643-10 PMC3088143

[45] Higazi DR, Fearnley CJ, Drawnel FM, Talasila A, Corps EM, et al (2009) Endothelin-1-stimulated InsP3-induced Ca^2+^ release is a nexus for hypertrophic signaling in cardiac myocytes. Mol Cell 33: 472–482. 19250908 10.1016/j.molcel.2009.02.005

[46] López JR, Espinosa R, Landazuru P, Linares N, Allen P, et al (2011) Dysfunction of diastolic [Ca^2+^] in cardiomyocytes isolated from chagasic patients. Rev Esp Cardiol 64: 456–462. 21511385 10.1016/j.recesp.2011.01.008

[47] De Souza AP, Tanowitz HB, Chandra M, Shtutin V, Weiss LM, et al (2004) Effects of early and late verapamil administration on the development of cardiomyopathy in experimental chronic *Trypanosoma cruzi* (Brazil strain) infection. Parasitol Res 92: 496–501. 14999469 10.1007/s00436-004-1080-1

[48] Colella M, Grisan F, Robert V, Turner JD, Thomas AP, et al (2008) Ca^2+^ oscillation frequency decoding in cardiac cell hypertrophy: role of calcineurin/NFAT as Ca^2+^ signal integrators. Proc Natl Acad Sci U S A 105: 2859–2864. 18287024 10.1073/pnas.0712316105 PMC2268550

[49] Bellio M, Liveira AC, Mermelstein CS, Capella MA, Viola JP, et al (1999) Costimulatory action of glycoinositolphospholipids from *Trypanosoma cruzi*: increased interleukin 2 secretion and induction of nuclear translocation of the nuclear factor of activated T cells 1. FASEB J 13: 1627–1636. 10463955 10.1096/fasebj.13.12.1627

[50] Kayama H, Koga R, Atarashi K, Okoyama M, Kimura T, et al (2009) NFATc1 mediates Toll-like receptor-independent innate immune responses during *Trypanosoma cruzi* infection. PloS Pathog 5: e1000514. 19609356 10.1371/journal.ppat.1000514 PMC2704961

[51] Tang M, Li J, Huang W, Su H, Liang Q, et al (2010) Proteasome functional insufficiency activates the calcineurin-NFAT pathway in cardiomyocytes and promotes maladaptive remodelling of stressed mouse hearts. Cardiovasc Res 88: 424–433. 20601385 10.1093/cvr/cvq217 PMC2972684

[52] Poteser M, Schleifer H, Lichtenegger M, Schernthaner M, Stockner T, et al (2011) PKC-dependent coupling of calcium permeation through transient receptor potential canonical 3 (TRPC3) to calcineurin signaling in HL-1 myocytes. Proc Natl Acad Sci U S A 108: 10556–10561. 21653882 10.1073/pnas.1106183108 PMC3127924

[53] Sugimoto T, Haneda M, Sawano H, Isshiki K, Maeda S, et al (2001) Endothelin-1 induces cyclooxygenase-2 expression via nuclear factor of activated T-cell transcription factor in glomerular mesangial cells. J Am Soc Nephrol 12: 1359–1368. 11423565 10.1681/ASN.V1271359

[54] Corral RS, Iñiguez MA, Duque J, López-Pérez R, Fresno M (2007) Bombesin induces cyclooxygenase-2 expression through the activation of the nuclear factor of activated T cells and enhances cell migration in Caco-2 colon carcinoma cells. Oncogene 26: 958–969. 16909108 10.1038/sj.onc.1209856

[55] Yiu GK, Toker A (2006) NFAT induces breast cancer cell invasion by promoting the induction of cyclooxygenase-2. J Biol Chem 281: 12210–12217. 16505480 10.1074/jbc.M600184200

[56] Cheng TH, Shih NL, Chen CH, Lin H, Liu JC, et al (2005) Role of mitogen-activated protein kinase pathway in reactive oxygen species-mediated endothelin-1-induced beta-myosin heavy chain gene expression and cardiomyocyte hypertrophy. J Biomed Sci 12: 123–l33. 15864745 10.1007/s11373-004-8168-6

[57] Miyatake S, Manabe-Kawaguchi H, Watanabe K, Hori S, Aikawa N, et al (2007) Prostaglandin E2 induces hypertrophic changes and suppresses alpha-skeletal actin gene expression in rat cardiomyocytes. J Cardiovasc Pharmacol 50: 548–554. 18030065 10.1097/FJC.0b013e318145ae2e

[58] Bai S, Kerppola TK (2011) Opposing roles of FoxP1 and Nfat3 in transcriptional control of cardiomyocyte hypertrophy. Mol Cell Biol 31: 3068–3080. 21606195 10.1128/MCB.00925-10 PMC3133389

[59] Rebsamen MC, Church DJ, Morabito D, Vallotton MB, Lang U (1997) Role of cAMP and calcium influx in endothelin-1-induced ANP release in rat cardiomyocytes. Am J Physiol 273: E922–E931. 9374678 10.1152/ajpendo.1997.273.5.E922

[60] Mukherjee S, Machado FS, Huang H, Oz HS, Jelicks LA, et al (2011) Aspirin treatment of mice infected with *Trypanosoma cruzi* and implications for the pathogenesis of Chagas disease. PLoS One 6: e16959. 21347238 10.1371/journal.pone.0016959 PMC3039660

[61] Freire-de-Lima CG, Nascimento DO, Soares MB, Bozza PT, Castro-Faria-Neto HC, et al (2000) Uptake of apoptotic cells drives the growth of a pathogenic trypanosome in macrophages. Nature 403: 199–203. 10646605 10.1038/35003208

[62] Ogletree ML (1987) Overview of physiological and pathophysiological effects of thromboxane A_2_ . FASEB J 46: 133–138. 2948837

[63] Chua CC, Hamdy RC, Chua BH (1996) Regulation of endothelin-1 production by a thromboxane A2 mimetic in rat heart smooth muscle cells. Biochim Biophys Acta 1313: 1–5. 8781542 10.1016/0167-4889(96)00042-0

[64] Jabbour HN, Sales KJ, Boddy SC, Anderson RA, Williams AR (2005) A positive feedback loop that regulates cyclooxygenase-2 expression and prostaglandin F2alpha synthesis via the F-series-prostanoid receptor and extracellular signal-regulated kinase 1/2 signaling pathway. Endocrinology 146: 4657–4664. 16081631 10.1210/en.2005-0804

